# The Codon Usage Code for Cotranslational Folding of Viral Capsids

**DOI:** 10.1093/gbe/evab089

**Published:** 2021-04-29

**Authors:** Rosa M Pintó, Albert Bosch

**Affiliations:** Enteric Virus Laboratory, Section of Microbiology, Virology and Biotechnology, Department of Genetics, Microbiology and Statistics, School of Biology, and Institute of Nutrition and Safety, University of Barcelona, Barcelona, Spain

**Keywords:** codon usage, translation selection, translation kinetics selection, protein folding, cotranslational folding, virus

## Abstract

Codon bias is common to all organisms and is the result of mutation, drift, and selection. Selection for the efficiency and accuracy of translation is well recognized as a factor shaping the codon usage. In contrast, fewer studies report the control of the rate of translation as an additional selective pressure influencing the codon usage of an organism. Experimental molecular evolution using RNA virus populations is a powerful tool for the identification of mechanisms underlying the codon bias. Indeed, the role of deoptimized codons on the cotranslational folding has been proven in the capsids of two fecal-orally transmitted picornaviruses, poliovirus, and the hepatitis A virus, emphasizing the role of the frequency of codons in determining the phenotype. However, most studies on virus codon usage rely only on computational analyses, and experimental studies should be encouraged to clearly define the role of selection on codon evolution.

##  

Codon bias, or the nonrandom usage of synonymous codons, is a common feature of most species in all domains of life. The particular codon usage of an organism reflects the evolutionary forces that have acted on its genome including mutation, genetic drift, and selection (Grantham et al. 1980; Hershberg and Petrov 2008; Sharp et al. 2010; Plotkin and Kudla 2011). It has been proposed that selection could favor preferred codons over rare codons to improve the efficiency and accuracy of the translation (i.e., translation selection), while mutational pressure and genetic drift would let minor codons to persist (Bulmer 1991; Duret 2002; Hershberg and Petrov 2008). However, there is also evidence of selection on rare or nonoptimal codons to control the ribosome traffic on the translating mRNA (i.e., translation kinetics selection). The underlying hypothesis is that tRNAs translating preferred codons would be abundant in the tRNA pool whereas tRNAs translating rare codons would be scarce. In this latter case, the longer time required for incorporating the scarce tRNAs into the ribosome A site would induce ribosome stalls slowing down translation elongation (Yang and Nielsen 2008; Komar 2009; Tuller et al. 2010; Plotkin and Kudla 2011; Wohlgemuth et al. 2013; Chaney and Clark 2015; Yu et al. 2015; Weinberg et al. 2016; Zhao et al. 2017). These ribosome stalls would play an essential role in the cotranslational folding of the nascent polypeptide by temporally separating the folding events, boosting the “favorable” and preventing the “unfavorable” interactions within the growing peptide (Yang and Nielsen 2008). An elegantly designed experiment demonstrated that the rate at which a nascent protein exits from the ribosome can specify the folded structure of a protein (Sander et al. 2014). This theory has been proposed as the codon usage “code” for protein folding (Yu et al. 2015; Liu 2020; Liu et al. 2021).

Several works have proven the role of nonoptimal codons in protein folding, including the *Neurospora* FREQUENCY protein (Zhou et al. 2013) and the *Drosophila* PERIOD protein (Fu et al. 2016), both essential for the circadian clock function. In humans, one of the most paradigmatic examples of how a silent mutation may influence the cotranslational folding and function of a protein is the synonymous single-nucleotide polymorphisms (SNPs) of the Multidrug Resistance 1 (MDR1) gene, which alters the substrate and inhibitor binding sites of its P-glycoprotein product (Kimchi-Sarfaty et al. 2007). Remarkably, synonymous SNPs have been correlated with human diseases in genome-wide association studies, but the underlying mechanisms have only been identified in a few of them (Chamary and Hurst 2009; Chen et al. 2010; Sauna and Kimchi-Sarfaty 2011). Genome projects generate massive amounts of sequences which may be comparatively analyzed using population genetics statistical models to detect footprints of selection (Kreitman 2000; Excoffier and Heckel 2006; Booker et al. 2017). However, these models do not usually consider selection for the efficiency, accuracy, and kinetics of translation. Hopefully, an alternative could arise from experimental evolution studies.

RNA viruses are a powerful tool for experimental evolution owing to their great evolutionary potential, which results from their very short generation time, their large population size, and their error-prone replication. RNA viruses exist as complex mutant swarms which are the target of selection (Domingo et al. 2012). The same molecular evolutionary rules apply to all organisms, from viruses to cells, but the relative weight of each driving process may vary between them. Different selective pressures may shape the codon usage of RNA viruses. For instance, viruses have evolved to very low CpG and UpA dinucleotide contents to evade antiviral cell responses, which in turn could influence their codon composition (Atkinson et al. 2014). Secondary structures in the genomic RNA, with functional roles through the interaction with viral or cellular proteins, could also constraint the codon usage (Gumpper et al. 2018), and finally yet importantly selection for an efficient and regulated translation may shape codon usage. An interesting example of a combined action of selection for translation efficiency and virus overcome of the cellular antiviral action is the codon usage of the avian influenza H3N2 virus PB1 mRNA, which is skewed towards interferon-altered human tRNA pools contributing to its codon bias (Smith et al. 2018).

Viruses are not able to synthesize tRNAs, hence they depend on the cellular tRNA pools for their own translation. Excessive codon usage similarity between virus and hosts may impede host translation, inducing a deleterious effect, and consequently, viruses have evolved to an optimal range of codon usage bias (Chen et al. 2020). Additionally, viruses may be able or not to inhibit the cellular protein synthesis, that is, the host shut off, and this will have critical implications on tRNA availability. Assuming translation efficiency as the main selective pressure shaping the codon composition, a codon usage in the optimal range with the host should be expected in viruses able to shut down the protein synthesis, like poliovirus (PV), while a more deviated codon usage should apply in viruses unable to do so, like hepatitis A virus (HAV) (Pintó et al. 2018). In this latter example, selection for a slow translation elongation has been proven to be the main selection for its deviated codon usage. In experimental evolution studies, HAV was adapted to grow in the presence of actinomycin D, which specifically inhibits the cellular DNA-dependent RNA polymerase with no effects on the viral RNA-dependent RNA polymerase, and evolved with an initial increase of nonoptimal codons in the capsid coding region and thereafter with an increase of optimal codons (Aragonès et al. 2010; Costafreda et al. 2014). These codon adjustments induced significant changes in the rate of translation (D'Andrea et al. 2019), and in turn in capsid features such as the antigenic structure, the physical stability, and the uncoating efficiency (Costafreda et al. 2014; D'Andrea et al. 2019). Similarly, adaptation of PV to conditions of suppression of chaperon activity promoted the selection of clusters of codon-deoptimized mutations, which may assist the cotranslational folding (Geller et al. 2018). In both cases, there were significant increases in the diversity of the mutant swarms, and, although the selected genotypes mostly harbored few or even single mutations, they gave rise to new translation phenotypes that in turn increased the diversity of capsid phenotypes. PV uses chaperon Hsp90 to support capsid folding (Geller et al. 2012), while HAV does not require this chaperon and instead uses deoptimized codons for the control of the cotranslational folding (Aragonès et al. 2010; Costafreda et al. 2014; D'Andrea et al. 2019). It can thus be concluded that an efficient translation rate requires a tradeoff with an efficient cotranslational folding, which may be achieved by chaperon action or by selecting codon deoptimization at specific sites. The noncoding function of codons has been defined as the code within the genomic code (fig. 1).

**Fig. 1.**
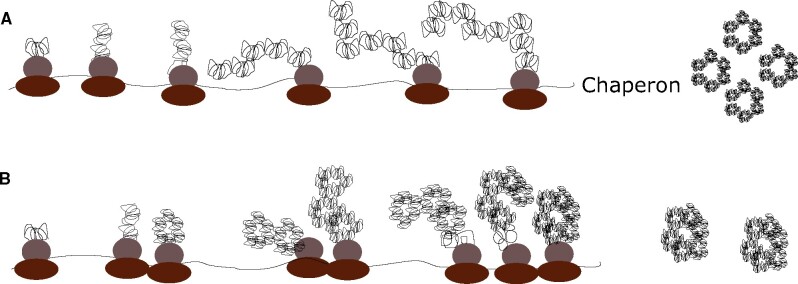
General mechanism of the potential impact of codon deoptimization on protein folding. (A) The use of abundant codons ensures a rapid an efficient translation with a high production of protein at the cost of absence of cotranslational folding, instead chaperons assist the protein folding. This is the model for the poliovirus capsid folding. (B) The occurrence of clusters of rare codons and the associated ribosome stalls locally slows down the kinetics of translation promoting the cotranslational folding at the cost of a low production of proteins. This is the model for the hepatitis A virus capsid folding.

There are still many questions to be resolved. Has the essential role of deoptimized codons in the control of cotranslational folding a relationship with the capsid symmetry and hence may be extended to other icosahedral capsids, or is it rather more related with aspects of the virus biology such as the mode of transmission? The fecal–oral route of transmission, used by PV and HAV, involves long periods outside the body host requiring highly stable capsids. In contrast, the arthropod-borne viruses, which have a broad spectrum of hosts and are transmitted by blood-feeding bites with no extra body phases, have evolved to codon usages resembling either their natural invertebrate hosts, when the vertebrates act as accidental dead-end hosts (bunyaviruses, rhabdoviruses, and reoviruses), or both invertebrate and vertebrate hosts (alphaviruses, flaviviruses, and orthomyxoviruses) when virus replication in vertebrates results in high-titer viremia (Velázquez-Salinas et al. 2016). In this latter scenario, virus transmission occurs through mosquito or tick bites, and to ensure transmission selection for an efficient translation would have contributed to shape their codon usage which would be in accordance with their capacity to induce the cellular shutoff (Fros and Pijlman 2016; Roth et al. 2017). Similarly, it has been described that viruses with a broad range of hosts have a worse codon usage match with their hosts than viruses with a narrow range of hosts (Tian et al. 2018) and that vector-borne viruses show lower codon bias than aerosol-transmitted viruses (Jenkins and Holmes 2003). Viral fitness is usually measured through the level of virus replication, but the efficient transmission is also a major factor with a critical impact in the overall fitness (Wargo and Kurath 2012). Codon usage may play a role in the mode of transmission of viruses, either facilitating the translation efficiency or controlling the capsid cotranslational folding.

It is important to emphasize the need to go one step further in identifying the underlying mechanisms of the codon usage bias in the different viruses, rather than simply describing their codon composition and comparing it with the host codon usage. Although there are outstanding studies showing selection for translation efficiency, that is, through the use of optimal codons and codon pairs, selection for the regulation of the translation rate, that is, through the right combination of optimal and nonoptimal codons, and selection for escaping the antiviral cell response, that is, through the low CpG and UpA contents, as mechanisms contributing to codon bias (Walsh et al. 2020; Burns et al. 2006; Mueller et al. 2006; Coleman et al. 2008; Mueller et al. 2010; Lauring et al. 2012; Tulloch et al. 2014; Martinez et al. 2016; Pintó et al. 2018; D'Andrea et al. 2019; Mueller et al. 2020), many others are merely descriptive. For instance, the total number of publications found in the PubMed archive in the period 2000–2020 using the terms RNA+Virus+Codon+Usage was of 469, adding the term translation was of 153, and adding the term folding was of only 18, and corresponding most of these references to genomic comparative analysis. It is essential to determine the type of selection pressures contributing to the codon bias of each virus under study. The topics of selection for the translation efficiency and selection for the codon-driven translation kinetics may take advantage of experimental evolution in the framework of the new advances in the analysis of tRNA abundance and heterogeneity, which is host and cell specific. Finally, in the case of the codon-driven cotranslational folding, it is necessary to know how universal it is, and to what extent the codon frequency dictates the phenotypic landscapes of the protein folding of the mutant swarms in tRNA changing environments (hosts and cell types, shut off, chaperons, interferon, temperature, etc.). Robustness in the different conditions could anticipate the evolvability of virus populations. The potential constraints or benefits that the codon composition of a virus population may impose for the adaptation to new hosts could contribute to identify potential zoonotic spillovers between very distant species. A deep understanding of the codes within the genomic code would contribute to advancements in the field of virus evolution.
